# Retraction: Nintedanib, a triple tyrosine kinase inhibitor, attenuates renal fibrosis in chronic kidney disease

**DOI:** 10.1042/CS20170134_RET

**Published:** 2026-06-03

**Authors:** Shougang Zhuang, Feng Liu, Li Wang, Hualin Qi, Jun Wang, Yi Wang, Wei Jiang, Liuqing Xu, Na Liu

**Affiliations:** 1 Department of Nephrology, Shanghai East Hospital, Tongji University School of Medicine, Shanghai, China; 2 Department of Medicine, Rhode Island Hospital and Alpert Medical School, Brown University, Providence, RI, USA

**Keywords:** folic acid, renal fibrosis, renal interstitial fibroblasts, transforming growth factor β1, unilateral ureteral obstruction

This article is being retracted from *Clinical Science* at the request of the authors following receipt of a notification from a reader, alerting the Editorial Office to multiple similarities within the data of this article. Specifically, this includes:

- The Figure 4A p-FGFR1 bands appear highly similar to the Figure 6A p-NFkB bands
- The Figure 2A B-actin bands appear highly similar to the Figure 10A NFkB bands
- In Figure 10A, the 4th and 6th p-Smad3 bands appear to be highly similar to each other, as do the 5th and 10th bands.
- In Figure 5A, the 3rd, 4th and 5th Src bands appear highly similar to the final three Src bands
- In Figure 5A, the 1st and 2nd p-Lck bands appear highly similar to the 5th and 6th p-Lck bands.
- In Figure 11D, the 1st and 2nd Collagen I bands appear highly similar to the 3rd and 4th Collagen I bands.

The authors were unable to provide a portion of the original data as it has been lost and is no longer available. Repeated experimental data was provided; however, the loss of the original data prevents the authors from adequately addressing the reader's concerns. The authors stated that similarities arose from inappropriate raw data management and unintentional human errors during image processing, which led to inadvertent band duplications, and that there was no deliberate academic misconduct or malicious image reuse. The Editorial Board remains concerned about raw data provided for Figure 11.

The authors wish to retract the article. The Editor-in-Chief and Editorial Board agree with the retraction.

